# Preliminary validation of nCounter PanCancer immune profiling of FFPE slides and pbmc in CITN-05, a CITN study of the immunological effects of an IDO1 inhibitor in patients with ovarian carcinoma

**DOI:** 10.1186/2051-1426-3-S2-P84

**Published:** 2015-11-04

**Authors:** Lucas Dennis, Steven P Fling, Joseph M Beechem, Patrick Danaher, Leonard D'Amico, Mary Disis, Nathan Elliott, Melissa Geller, Celine Jacquemont, Steven Kussick, Richard Shine, Kunle Odunsi

**Affiliations:** 1 NanoString Technologies, Seattle, WA, USA; 2 Fred Hutchinson Cancer Research Center, Seattle, WA, USA; 3 UW School of Medicine Tumor Vaccine Group, Seattle, WA, USA; 4 University of Minnesota, Minneapolis, MN, USA; 5 Phenopath, Seattle, WA, USA; 6 Roswell Park Cancer Institute, Buffalo, NY, USA

## Background

The Cancer Immunotherapy Trials Network (CITN) is conducting a pilot study in patients with advanced epithelial ovarian, fallopian tube or primary peritoneal carcinoma to determine the extent to which a regimen of an IDO1 inhibitor that normalizes Kyn/Trp ratios might be immunotherapeutic and alter the tumor microenvironment, including the number and character of tumor infiltrating lymphocyte and the gene signature of infiltrating cells (PI: Kunle Odunsi). Here we describe preliminary comparative analyses of NanoString's nCounter gene expression technology, IHC (PhenoPath) and Flow cytometry (CITN Immune Monitoring Laboratory) using FFPE slides and PBMCs from treated subjects.

## Methods

Tumor biopsies, whole blood and Ficoll-gradient purified PBMCs were collected from study subjects at multiple study time-points. All subjects were treated for 2 weeks (Day 1 through D14) with IDO1 inhibitor, INCB024360 (Incyte). The number and percentage of PBMC subsets and T lymphocyte subsets were evaluated by multiparameter flow cytometry. Tumor biopsies were scored for lymphocyte (CD3 and CD8) infiltration by standard IHC. Gene expression from both PBMC and FFPE biopsies were evaluated using the nCounter PanCancer Immune Profiling panel that interrogates 770 immune-related genes and associated controls using ~60,000 cell lysates for PBMC and 300ng of total extracted RNA from 4-micron FFPE sections from tumor biopsies.

## Results

IHC identified differential degrees (high, moderate, low) of lymphocyte infiltration in FFPE biopsies. A strong correlation to IHC results was observed in nCounter RNA-based gene expression of neighboring FFPE sections. The majority of PBMC and T lymphocyte subsets measured by flow also trended positively with gene-expression data, both across study participants and across collection time-points for each patient. In addition to correlative association with flow and IHC, gene expression profiling identified many differentially expressed targets between trial subjects and across trial time-points.

## Conclusions

These data establish the nCounter PanCancer Immune Profiling panel as a valuable investigative tool in immunotherapy trials. The strong correlative associations between protein expression by flow and IHC with gene expression profiling by nCounter PanCancer Immune Profiling or other methods may ultimately expedite the identification of vital and clinically relevant biomarkers.

